# Molecular Mechanisms Underlying Peritoneal EMT and Fibrosis

**DOI:** 10.1155/2016/3543678

**Published:** 2016-01-31

**Authors:** Raffaele Strippoli, Roberto Moreno-Vicente, Cecilia Battistelli, Carla Cicchini, Valeria Noce, Laura Amicone, Alessandra Marchetti, Miguel Angel del Pozo, Marco Tripodi

**Affiliations:** ^1^Department of Cellular Biotechnologies and Hematology, Section of Molecular Genetics, Sapienza University of Rome, Viale Regina Elena 324, 00161 Rome, Italy; ^2^Integrin Signaling Laboratory, Cell Biology & Physiology Program, Cell & Developmental Biology Area, Centro Nacional de Investigaciones Cardiovasculares Carlos III (CNIC), Melchor Fernández Almagro 3, 28029 Madrid, Spain

## Abstract

Peritoneal dialysis is a form of renal replacement alternative to the hemodialysis. During this treatment, the peritoneal membrane acts as a permeable barrier for exchange of solutes and water. Continual exposure to dialysis solutions, as well as episodes of peritonitis and hemoperitoneum, can cause acute/chronic inflammation and injury to the peritoneal membrane, which undergoes progressive fibrosis, angiogenesis, and vasculopathy, eventually leading to discontinuation of the peritoneal dialysis. Among the different events controlling this pathological process, epithelial to mesenchymal transition of mesothelial cells plays a main role in the induction of fibrosis and in subsequent functional deterioration of the peritoneal membrane. Here, the main extracellular inducers and cellular players are described. Moreover, signaling pathways acting during this process are elucidated, with emphasis on signals delivered by TGF-*β* family members and by Toll-like/IL-1*β* receptors. The understanding of molecular mechanisms underlying fibrosis of the peritoneal membrane has both a basic and a translational relevance, since it may be useful for setup of therapies aimed at counteracting the deterioration as well as restoring the homeostasis of the peritoneal membrane.

## 1. Introduction

Peritoneum is a serosal membrane that forms the lining of the abdominal cavity. It is composed of a continuous monolayer of cells of mesodermal origin, the mesothelial cells (MCs). MCs have an epithelial-like cobblestone shape and cover a submesothelial region constituted of a thin layer of connective tissue composed mainly of bundles of collagen fibers with few fibroblasts, mast cells, macrophages, and vessels [1]. Peritoneum supports the abdominal organs and serves as a conduit for their blood vessels, lymph vessels, and nerves. Between parietal peritoneum, covering the abdominal wall, and visceral peritoneum, covering abdominal viscera, resides the peritoneal cavity, a virtual space filled of scarce interstitial fluid. This fluid facilitates peristaltic movements of abdominal viscera. Moreover, peritoneum is relevant for the control of local and intestinal immunity due to leukocyte recirculation [2].

Peritoneal membrane can be used as a dialysis membrane in therapeutic procedures for the treatment of end-stage renal disease, as an alternative to classical hemodialysis procedure [3]. Currently, peritoneal dialysis (PD) accounts for more than 10% of all forms of renal replacement therapy worldwide [3]. During PD, the peritoneal membrane (PM) acts as a permeable barrier across which ultrafiltration and diffusion take place [4]. Continual exposure to hyperosmotic, hyperglycemic, and acidic dialysis solutions, mechanical stress connected to dwelling practice, and episodes of catheter complications (including peritonitis and hemoperitoneum) may cause acute and chronic inflammation and injury of the PM. In these conditions, peritoneum undergoes progressive fibrosis, angiogenesis, and vasculopathy, eventually leading to discontinuation of PD.

A main role in the induction of peritoneal fibrosis during exposure to PD fluids is played by the epithelial to mesenchymal transition (EMT) of mesothelial cells (MCs), named more properly mesothelial to mesenchymal transition (MMT) [5]. The EMT represents a complex phenomenon of cellular transdifferentiation that converts the epithelial phenotype into a mesenchymal one, with loss of cell polarization, disassembly of adherent and tight junctions, and, conversely, the acquisition of fibroblastic shape and ability to invade. The EMT process characterizes physiological (i.e., organogenesis, development, wound healing, and regeneration) as well as pathological (i.e., fibrosis, tumor progression, and metastasis) processes [6].

In this review, we highlight current knowledge about cellular players and molecular mechanisms triggering PM fibrosis. In particular, we summarize the evidence supporting the involvement of EMT in this phenomenon, with emphasis on the response to signals delivered by TGF-*β* family members and by Toll-like/IL-1*β* receptors, molecules playing a main role in EMT induction in the PM.

## 2. Induction of Fibrosis during PD

During practice of PD, modifications of the PM occur virtually in all patients. Signs of peritoneal fibrosis are detected in 50% to 80% of patients within one to two years on PD [7]. In many cases, the peritoneal alterations are limited and result in a simple peritoneal sclerosis (SPS). SPS is characterized by increased thickness of the submesothelial space, increased angiogenesis with hyalinizing vasculopathy, and presence of denuded areas with loss of MCs. In this form, the entity of fibrosis is generally limited; it correlates with the length of exposure to PD fluid and is reversible when PD is interrupted [8]. In some cases, the patients develop encapsulating peritoneal sclerosis (EPS), which is a potentially deadly form of peritoneal fibrosis characterized by severe peritoneal thickening, inflammation, calcifications, and fibrin deposits [9]. Fibrosis may progress even if the patient switches to another form of renal replacement and may evolve in visceral encapsulation with episodes of bowel obstruction. The pathogenesis of EPS is debated: it is uncertain whether EPS evolves as a progression of SPS or whether it is a primitive form of sclerosis [10].

## 3. Cellular Players of Peritoneal Fibrosis

When exposed to a wide range of exogenous or endogenous inflammatory/profibrotic stimuli, both cellular components of peritoneum (MCs, macrophages, mast cells dermal fibroblasts, endothelial cells, and resident macrophages) and other elements of innate and adaptive immunity actively participate in the induction of the inflammatory response.

In the case of acute peritonitis, a first wave of neutrophils recruited by chemoattractants of bacterial origin (LPS) is progressively replaced by a population of mononuclear cells, composed of monocytes/macrophages and lymphocyte subsets [11]. In this context, IL-6 plays a main role. IL-6 soluble receptor (s-IL-6R) shed by neutrophils favors, through a process called “transsignaling,” the production of chemokines, including CXCL8 and CCL2, able to recruit mononuclear cells [12]. Besides directing leukocyte recruitment to inflamed peritoneum, these chemokines directly target MCs and other components of the peritoneum, and their inhibition may limit peritoneal fibrosis [13, 14].

Both MCs and peritoneal macrophages respond to the first neutrophil wave and the secondary mononuclear cell predominance producing a wide array of inflammatory cytokines such as IL-1*β*, TNF-*α*, IL-6, and other proinflammatory mediators (chemokines, endogenous Toll-like receptor (TLR) ligands) [15, 16]. At the same time, molecules with anti-inflammatory activities, such as IL-10 and TGF-*β*, are released in the peritoneal cavity. Mast cells have been demonstrated to play a role in kidney fibrosis through production of tryptase and chymase [17]. Moreover, their number is increased in peritoneum, and they produce fibrogenic factors in a model of peritoneal fibrosis in rats [18].

Besides components of the innate immunity, more recent studies performed using murine models demonstrated a main role of T helper 1 (Th1) cell response and of T lymphocytes expressing IL17A [19–21]. In these conditions, the presence of IL-6 is particularly relevant since it may shape the immune response in subacute-chronic conditions. IL-6, in combination with TGF-*β*, is the main cytokine involved in the T helper 17/regulatory T (Th17/Treg) balance [21]. The predominance of IL-6 favors the generation of Th-17 lymphocytes, which produce inflammatory cytokines. On the other hand, TGF-*β* in the absence of IL-6 promotes the Treg lineage, producing the anti-inflammatory cytokine IL-10. The modulation of the expression of these cytokines through biologic antibodies or recombinant cytokines is an attracting field for the design of new therapies aimed at counteracting peritoneal EMT and fibrosis [20, 22, 23]. The presence of proinflammatory and profibrotic cytokines determines the following: (i) the aberrant production of extracellular matrix (ECM) proteins, such as Fibronectin (FN) and type I collagen (Coll) in the submesothelial stroma, (ii) an unbalanced ratio between procoagulant and anticoagulant factors (plasminogen activator inhibitor- (PAI-) 1/plasmin), and (iii) an altered production of glycosaminoglycans and proteoglycans constituting the extracellular fluid, which are responsible for the lubrication of the two peritoneal sheets (parietal and visceral) [16]. In particular, during PD, expression of high molecular weight hyaluronan and decorin is reduced, whereas low molecular weight hyaluronan and versican are induced [24, 25]. Differential expression of glycosaminoglycans and proteoglycans has pathogenic significance, since decorin may modulate the bioactivity of TGF-*β*1, thus directly affecting the entity of peritoneal fibrosis, whereas hyaluronan fragments have been shown to induce multiple signaling cascades, cytokine secretion, and matrix metalloproteases (MMP) activity [26].

Moreover, neoangiogenesis detected in the peritoneal stroma is due mainly to the effect of VEGF production, whose levels correlate with alterations in transport rate [27].

## 4. EMT of MCs as a Main Cause of Peritoneal Fibrosis

Besides contributing to production of cytokines and other soluble factors relevant to sustaining and modulating the inflammatory reaction, MCs through EMT play a central role in the alterations of the PM leading to fibrosis. A seminal study by Yáñez-Mó and collaborators first demonstrated that EMT of MCs plays a role in the onset of fibrosis in PD patients [5]. This study was followed by others, where with the help of animal models the main characteristics of MC EMT were elucidated [23, 28–30].

Not all the features of MC EMT parallel those of epithelial cells: due to their mesodermal origin, and differently from “true” epithelia, such as hepatocytes or keratinocytes, MCs coexpress in basal conditions epithelial and mesenchymal markers. This may explain their enhanced plasticity. With respect to epithelial markers, these cells express high amount of epithelial cytokeratins, such as cytokeratin 8-18, and proteins of tight and adherens junctions, such as junctional adhesion molecule 1 (JAM1) and zonula occludens-1 (ZO-1). E-cadherin has a peculiar distribution in MCs, since it is expressed both in membrane and in cytoplasm [28]. Similarly to mesenchymal cells, MCs express constitutively the intermediate filaments vimentin and desmin [2, 5].

The exposure to inflammatory/profibrotic stimuli leads to a rapid E-cadherin downregulation, which parallels an induction of N-cadherin (cadherin switch). While E-cadherin expression is rapidly downregulated, the expression of cytokeratins is only gradually lost; thus, transdifferentiated cells can maintain for long time trace of their origin.

E-cadherin downregulation parallels the induction of Snail, a master factor of EMT, directly inhibiting the E-cadherin transcription [5]. At the same time, the expression of the specific mesothelial differentiation factor Wilms tumor 1 (WT1) is reduced [31].

While epithelial features are lost, MCs rapidly gain expression of molecules related to EMT, such as *α*-SMA and FSP1. Moreover, MCs produce high levels of PAI-1, which plays a role in fibrin deposits and fibrosis. Also, ECM molecules such as FN and Coll are produced, as well as metalloproteases MMP2 and MMP9, which degrade the ECM favoring MCs invasive activity [32, 33].

The expression of *α*-SMA and FSP1 by MCs makes them a conceivable main source of myofibroblasts, the cells endowed with ability to contract the ECM and considered mostly responsible for the abnormal production of ECM in fibrosis of all organs [34, 35]. The myofibroblast in a fibrotic organ is thought to emerge by the activation and modification of different cellular components: lineage tracing studies demonstrated that epithelia may take a role in the generation of myofibroblasts in fibrotic kidney and lung, whereas endothelium is relevant in the production of myofibroblasts in heart through a process called Endothelial to Mesenchymal Transition (EndMT) [36–38].

Myofibroblasts are absent in normal peritoneum, whereas they are found in PM of patients undergoing PD or in mice exposed to PD fluids [28, 29]. In mice exposed to PD fluid, it has been demonstrated that myofibroblasts found in the PM have different origins, including resident dermal fibroblasts, endothelial cells, bone marrow derived cells, and MCs [23]. Cells coexpressing cytokeratin (as MCs marker) and FSP1 or *α*-SMA (as myofibroblast markers) invade the submesothelial stroma, where they take a role in regulating mesothelial thickness, angiogenesis, leukocyte chemotaxis, and perturbation of ultrafiltration function [23, 28, 39].

Interestingly, once the EMT-inducing stimuli have been removed, transdifferentiated MCs tend to maintain their “mesenchymal” state (Strippoli, unpublished). This observation, essentially based on* in vitro* studies, deserves further analyses since EMT reversal, the phenomenon named Mesenchymal to Epithelial Transition (MET), is a mechanism of peritoneal recovery that may take place* in vivo*. During mechanical or biochemical stresses including PD, areas of PM become devoid of cells. In these conditions, floating MCs (that have suffered a “bona fide” EMT) may reattach and restore cell-to-cell contacts, undergoing MET [40]. Interestingly, these mesenchymal-like MCs may be isolated from PD fluids and cultured* in vitro*. Upon exposure to soluble factors or inhibition of specific pathways, they may partially reacquire an epithelial-like state [32, 41, 42].

## 5. Extracellular Inducers of Fibrosis

### 5.1. Factors Related to Dialysis Fluid Bioincompatibility and Uremia

Nonphysiologic characteristics of conventional PD fluid, such as hypertonicity, the presence of high concentrations of glucose and lactate, and acidic pH, are associated with production of inflammatory cytokines and other molecules. High glucose (HG) itself may induce a proinflammatory and profibrotic reaction [43]. Many lines of evidence suggest that the local injury induced by classical glucose-based PD fluids is mediated, at least in part, by the presence of glucose degradation products (GDPs) and by the acidic pH. GDPs through the formation of advanced glycation-end products (AGEs) may stimulate the production of extracellular matrix components (ECM) as well as the synthesis of profibrotic and angiogenic factors [11, 44]. Several studies have demonstrated the appearance of AGEs in the peritoneal effluents of PD patients, which correlated with the time on PD treatment. Biopsy studies have confirmed the accumulation of AGEs in the peritoneal tissues of PD patients. AGEs accumulation is associated with fibrosis and ultrafiltration dysfunction [11]. AGEs accumulate also in condition of prolonged hyperglycemia not related to PD practice, such as in patients with diabetes mellitus and during kidney diseases [45]. Uremia* per se* is sufficient for inducing fibrosis in peritoneum, which is further increased when uremic patients undergo PD [46–48]. Various uremic solutes have been characterized. Among them, indoxyl sulfate, a derivative from tryptophan, plays a role in inducing fibrosis in kidney via ROS generation and TGF-*β* production [49].

The use of solutions with neutral pH and with low content of GDPs may represent a potential strategy to attenuate some of the PD-related adverse effects.

### 5.2. Release of Bacterial Molecules and TLR Ligands

Besides factors related to bioincompatibility of PD fluid, other inflammatory stimuli are linked to events connected to catheterization, such as hemoperitoneum and peritonitis. Both Gram-positive and Gram-negative bacteria may play a role in PM injury during PD. Administration of LPS in mice peritoneum induces production of inflammatory cytokines and chemokines and PM damage [50, 51].

Besides inducing a response mediated by Toll-like receptor (TLR) 4, LPS may take a role in the release of HMGB1, ubiquitous nonhistone nuclear protein capable of activating innate immune response through engagement of TLRs [50]. MCs may sense bacterial pathogens also through cytoplasmic Nod-like receptors, which may also induce production of inflammatory cytokines and chemokines [52]. Also, fragments of hyaluronic acid released during inflammation can induce EMT in MCs through engagement of TLRs [16, 53, 54].

### 5.3. TGF-*β*1 and Other Cytokines

Among different cytokines and inflammatory mediators elicited during peritoneal inflammation, TGF-*β*1 is considered the main mediator of peritoneal fibrosis. TGF-*β*1 belongs to a family of growth factors that includes TGF-*β*s, activins, and bone morphogenic proteins (BMPs) [55, 56]. Among all the members, TGF-*β*1 and BMP-7 are key determinant factors in peritoneal cell plasticity and, in particular, the predominance of one or the other may determine the epithelial or mesenchymal phenotype of MCs. TGF-*β*1 is present in fluids from patients undergoing PD and its levels correlate with deterioration of peritoneal membrane [57]. The role of TGF-*β*1 has been demonstrated in animal models, in which the intraperitoneal injection of adenovirus carrying TGF-*β*1 gene induced a peritoneal fibrosis similar to that induced upon exposure to PD fluids [29]. In a mouse model of peritoneal fibrosis, TGF-*β*1 blocking peptides preserved the peritoneal membrane by PD fluid induced damage [23].

The epithelial-like phenotype of MCs, together with their metastability and plasticity, is the result of a balance between constitutively secreted factors (including TGF-*β*1 and its “counteracting” BMP7, whose expression has been shown to interfere with fibrogenic activity of TGF-*β*1) and other extracellular stimuli [41, 43, 58]. In this regard, BMP7/TGF-*β*1 balance may be altered by other cytokines produced during the inflammatory response. For example, CTGF is produced in response to TGF-*β*1 and inhibits BMP7 effects [59]. Also, gremlin concentration in the peritoneal effluent correlated with measures of peritoneal membrane damage and may modulate BMP7-mediated effects [60]. HGF may stabilize the epithelial phenotype inhibiting EMT in MCs [43]. EGF which supports the epithelial state in some experimental systems, fostering EMT, and invasion in others has been recently demonstrated to promote peritoneal fibrosis through a cross talk with TGF-*β* mediated signals [61]. Besides these inflammatory mediators, many other cytokines that cooperate in peritoneal EMT/fibrosis induction (i.e., IL-1*β*, IL-6, and TNF-*α*, VEGF, and endothelin-1) are secreted by MCs and other cells in peritoneum [16, 62].

## 6. Molecular Mechanisms of EMT and Fibrosis

The complexity of proteome reprogramming occurring during EMT-MET dynamics, often involving dysregulation of specific differentiation processes, suggests the occurrence of cell-specific molecular mechanisms driving EMT and fibrosis [63]. Moreover, the molecular mechanisms driving EMT in different processes (i.e., embryogenesis or tumor) may be different even in the same cell type. In the case of the MCs, only a limited number of studies focused on the understanding of molecular mechanisms underlying EMT induction, compared to other experimental systems. Cell specificity is evident in the case of HGF, a cytokine which is generally considered a “pro-” EMT factor, whereas in MCs it has an anti-EMT activity [43, 64]. To complicate the picture, the same pathway may induce both pro-EMT and anti-EMT effects depending on the experimental conditions. This is the case of p38 MAPK, which is a main inducer of inflammatory cytokine production, thus potentially favoring EMT, but also promoting E-cadherin expression and the epithelial-like phenotype in MCs [65, 66]. The study of the role of a specific signaling pathway has been often performed using pharmacological inhibitors. Although the interpretation of the results obtained should be carefully evaluated considering the “caveat” of a possible lack of specificity, the “pharmacological approach” is especially relevant from a translational point of view, since it is possible to hypothesize the design of pharmacological treatments designed to specifically preserve or recuperate the PM homeostasis in PD patients.

### 6.1. TGF-*β*1 Induced Signaling Pathways

**Figure 1:**
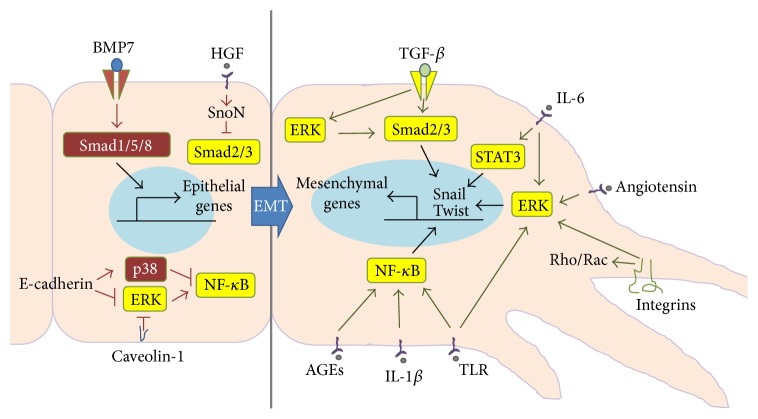
The epithelial/mesenchymal status of MCs is due to the balance of signals delivered by multiple receptors. Stimuli promoting EMT are delivered by TGF-*β* in cooperation with inflammatory cytokines and other mediators such as IL-1*β*, IL-6, TLR ligands, AGEs, and angiotensin. Smad2/3 pathway plays a main role in combination with ERK1/2 and NF-*κ*B pathway and all converge on the expression of Snail, the master gene of EMT. Integrin activation promotes the induction of conformational changes and the invasivity of MCs. On the other hand, signals delivered by BMP7 and HGF favor the epithelial phenotype through the activation of Smad1/5/8 and the inhibition of the Smad2/3 signaling. Also, signals delivered by cell-to-cell confluency (E-cadherin omotypic junctions) may lead to predominance of p38 MAPK over ERK1/2 and to the inhibition of NF-*κ*B activity. Caveolin-1 organizes signaling platforms favoring the stability of membrane receptors and inhibiting the Ras/MEK/ERK1/2 pathway.

With TGF-*β*1 being the main factor controlling fibrosis in all organs, it is not a wonder whether the main signaling pathways responsible for EMT induction in MCs are induced by this cytokine. Signaling pathways induced by TGF-*β*1, as well as TGF-*β* family members, are generally divided into Smad-dependent and Smad-independent ones. TGF-*β* factors signal via heterodimeric serine/threonine kinase transmembrane receptor complexes. The binding of the ligand to its primary receptor (receptor type II) allows the recruitment, transphosphorylation, and activation of the signaling receptor (receptor type I). Receptor type I of TGF-*β*1, or activin receptor-like kinase 5 (ALK5), is then able to exert its serine-threonine kinase activity phosphorylating Smad2 and Smad3. Receptor type I of BMP-7 (ALK3) phosphorylates instead Smad1, Smad5, and Smad8. Upon phosphorylation, they form heterodimers with Smad4, a common mediator of all Smad pathways [15, 55, 56, 67]. The resulting Smad heterocomplexes translocate into the nucleus where they bind directly to DNA and activate specific target genes (Figure 1). A third group of Smads composed of Smad6 and Smad7, called also inhibitory Smads, limit BMP-7- and TGF-*β*1-triggered Smad signaling, respectively, by preventing the phosphorylation and/or nuclear translocation of Smad2/3 or Smad1/5/8 complexes and by inducing their degradation through the recruitment of ubiquitin ligases [55, 56, 67].

The role of Smad3 signaling in TGF-*β*1 induced EMT and fibrosis is demonstrated* in vivo* in Smad3 knockout mice, which are protected from peritoneal fibrosis, show reduced collagen accumulation, and display attenuated EMT [39]. On the other hand, Smad2 may play an antagonistic role in the EMT process* in vivo*. Data in peritoneum are lacking; however Smad2 deficiency increases EMT in keratinocytes and hepatocytes [68, 69]. Despite their relevance in EMT/MET induction, the transcriptional activity of Smads alone is low, compared to other transcription factors: they display their activity when other transcription factors such as those from Snail, bHLH, or NF-*κ*B families are present [70].

Targeting Smad signaling by inhibitory Smad7 blocks EMT and reduces peritoneal fibrotic lesions [71]. Moreover, HGF and BMP-7 display their effect of EMT inhibition limiting Smad2/3 activity in MCs (Figure 1) [43, 58].

Indeed, HGF may interfere with TGF-*β*1 mediated EMT inducing the expression of the transcriptional corepressor SnoN, which interacts with activated Smad2/4 complex and blocks the expression of Smad-dependent genes [72]. BMP-7 inhibitory effect on EMT is dependent on the activation of Smad1/5/8 proteins that counteract TGF-*β* activated Smad2/3 activity [58].

MCs constitutively express BMP-7 and display basal activation of Smad1/5/8, which contribute to the maintenance of the epithelial-like phenotype. EMT induction by TGF-*β*1 results in BMP-7 downregulation and inactivation of BMP-7-specific signaling [58].

**Figure 2:**
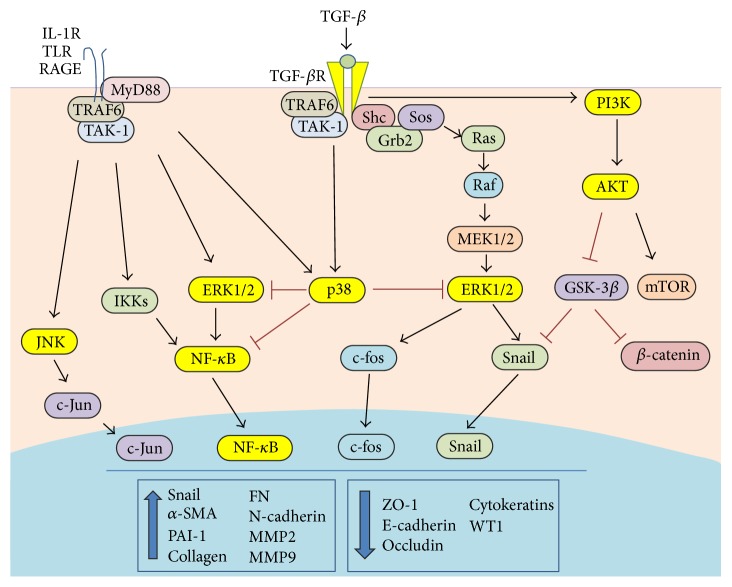
Cooperation between signals delivered by TGF-*β* and Toll-like/IL-1*β* receptors in the EMT of MCs. TGF-*β* delivers pro-EMT signals inducing the Smad2/3 (not described in this figure) and the non-Smad pathways, composed of MEK/ERK1/2 and PI3K pathways. IL-1*β* and TLR ligands activate redundant pathways leading to activation of NF-*κ*B and ERK1/2. Also, pathways able to limit EMT induction, such as p38, are induced at the same time. Smad2/3 acted as transcription factor in combination with Snail, NF-*κ*B, and AP-1 to induce the EMT program.

TGF receptors may also activate signaling pathways independently of Smads (Figure 2) [55, 73]. Mitogen activated protein kinases (MAPKs), Rac and Rho GTPases, phosphatidyl inositol 3 kinase (PI3Kinase)/Akt pathways are relevant in different cellular function elicited by TGF-*β*1 in different EMT experimental systems. TGF-*β* induced MEK/ERK1/2 is particularly relevant in EMT and fibrosis [74]. TGF-*β* RI may induce ERK1/2 pathway through tyrosine phosphorylation of ShcA adaptor protein and subsequent recruitment of Grb2/Sos complex [75]. In MCs, inhibition of the MEK/ERK1/2 pathway limited EMT induced by TGF-*β*1 in combination with IL-1*β*, a cytokine mimicking an inflammatory stimulus, and induced MET in MCs from PD patients that had undergone EMT* in vivo* [32]. Moreover, pharmacological inhibition of MEK/ERK1/2 pathway rescued E-cadherin and ZO-1 altered expression, reduced fibrosis, and restored peritoneal function in mice exposed to PD fluids [28].

Interestingly, TGF*β* induced MEK/ERK1/2 pathways may alternatively enhance or limit Smad activities.

ERK1/2 may phosphorylate R-Smads in their linker region, thus inhibiting nuclear translocation and transcriptional activity [76]. More recently, it has been observed that ERK1/2 phosphorylation of the linker region of nuclear localized Smads resulted in increased half-life of C-terminal Smad2 and Smad3 phosphorylation and increased duration of Smad target gene transcription [77]. MEK/ERK1/2 pharmacological inhibition in MCs reduced Smad3 activity in luciferase assays, which correlated with reduced C-terminus Smad3 phosphorylation. Interestingly, in the same conditions Smad1/5 luciferase activity was increased, with increased C-terminus phosphorylation [28]. The intensity of MEK/ERK1/2 response can be modulated by intracellular factors. Caveolin-1, the principal marker of caveolae, plasma membrane specialized structures, limits the intensity of the EMT response through an effect on TGF-RI internalization or a direct effect on Ras/MEK pathway [28] (Figure 1).

TGF-*β*1 may induce p38 and JNK MAPK activation pathway through activation of TAK1 (TGF-*β* activated protein) [78]. Besides being a main driver of inflammation, p38 MAPK plays a role in the control of cell differentiation and apoptosis [65].

p38 is stably activated in quiescent MCs and, differently from ERK1/2, its activation levels are increased in conditions of cellular confluency in MCs [66]. p38 activity maintains E-cadherin expression in MCs and p38-mediated pathway modulates the mesenchymal conversion of MCs by a feedback mechanism based on the downregulation of ERK1/2, TAK-1/NF-*κ*B activities (Figure 1) [66]. JNK inhibition leads to the maintenance of E-cadherin expression and block of EMT, similarly to ERK1/2 inhibition [66, 79].

Besides p38 and JNK, TAK-1 is an activator of NF-*κ*B. NF-*κ*B inhibition may limit EMT-related events in MCs [32]. Having a wide effect on TGF-*β*1 induced pathways, it is not surprising that TAK-1 inhibition may induce EMT reversal in MCs from PD patients [42].

Among the non-Smad mechanisms involved in EMT, also PI3K/Akt pathway has been extensively studied [80]. PI3K activates Akt through phosphorylation at serine 473. Once activated, Akt has multiple actions including the activation of mammalian TOR complex 1 (mTORC1) and mTORC2 [80]. Both complexes are involved in different aspects of EMT and invasion and are sensible to prolonged treatment with rapamycin [81]. On the other hand, mTORC2 phosphorylates and activates Akt. Treatment with rapamycin abrogated transition response, such as induction of *α*-SMA expression, in Smad3 deficient mice [39]. Moreover, it induced stabilization of *β*-catenin, another factor implicated in EMT induction [80]. Interestingly, rapamycin inhibited in the same experimental system hypoxia-induced VEGF expression and angiogenesis [82].

Both Smad and non-Smad pathways converge on activation of Snail, the master factor of EMT. Snail is a direct inhibitor of E-cadherin expression [83]. Moreover, Snail inhibits the expression of other proteins associated with cell junctions, such as claudins and occludin, with knock-on effects on the expression of other proteins such as metalloproteinases, integrins, and ECM proteins [84]. Besides Snail, also Slug (Snail2), ZEB1-2, and members of the basic helix-loop-helix (bHLH) family, such as Twist and E47, play a role in repressing E-cadherin expression and inducing EMT. They often have some tissue specificity but similar mechanism of action [85]. Snail is strongly induced in MCs upon treatment with TGF-*β*1. Immunofluorescence analyses show that Snail has a distribution mainly nuclear: this suggests that mechanisms favoring cytoplasmic accumulation are probably inactive in MCs [28]. Inhibition of Smad3, MEK/ERK1/2, and NF-*κ*B results in reduced Snail expression in MCs [32, 39]. Compared to Snail, Slug is faintly induced in MCs treated with TGF-*β*1, whereas Twist is not induced by the same stimulus (Strippoli, unpublished). Interestingly, p38 inhibition parallels Snail inhibition in MCs, whereas induction of Twist is observed [66].

### 6.2. TLR Ligands Induced Signaling Pathways

During inflammatory EMT, signals elicited by mediators of inflammation cooperate with pathways elicited by activation of TGF-*β* (Figure 2). This is particularly relevant in the case of peritoneum, which may undergo episodes of acute inflammation as in the case of bacterial peritonitis due to catheter implantation. In the case of PD dysfunction induced by high glucose or the presence of AGEs, peritoneum undergoes subacute inflammatory alterations, but the molecular mechanisms of damage are often similar.

Human MCs express ligands for both Gram-positive and Gram-negative TLR ligands, such as TLR1, TLR2, and TRL5, but not TLR4 [86]. TLRs have a cytoplasmic signaling domain homologous to that of IL-1 receptor (IL-1R), called the Toll/IL-1R domain [87].

Ligand binding to Toll-like receptor (TLR)/IL-1R family members results in the association of MyD88 with the cytoplasmic tail of receptors; this then initiates the signaling cascade that leads to the activation of NF-*κ*B and MAPKs [87]. Besides IL-1 and TLR ligands, also signaling from AGEs to their receptors RAGEs converges on NF-*κ*B and MAPK pathways (Figure 2) [88]. Moreover, signals delivered by IL-1/TLR ligands affect signaling from other pathways relevant in EMT induction, such as IL-6. IL-1*β* induces IL-6, and this cytokine may amplify IL-1 response in macrophages and synovial fibroblasts [89].

In MCs, IL-1*β* is a much stronger inducer of NF-*κ*B response than TGF-*β*1, and their costimulation generates an additive response. Inhibition of NF-*κ*B blocks EMT induction upon TGF-*β*1/IL-1*β* costimulation and partially reverses* in vivo* EMT in MCs from PD patients [32]. In the same cells, NF-*κ*B nuclear translocation and transcriptional activity is enhanced by MEK-ERK1/2 pathway and is inhibited by p38 [66]. NF-*κ*B controls Snail and Twist expression and cooperates with Snail in inducing FN transcription [32, 90, 91]. Moreover, NF-*κ*B is a transcriptional inducer of cyclooxygenase-2 (COX-2), a main mediator of inflammation [88]. Inhibition of COX-2 with celecoxib resulted in reduced fibrosis and in partial recovery of ultrafiltration in mice exposed to PD fluids [92]. Interestingly, Twist is increased in MCs exposed to high glucose (probably due to NF-*κ*B activation)* in vitro* and in the PM of mice exposed to high glucose PD fluids, and it is linked to MMP9 to MMP9 production and MCs invasion [33].

Overall, it is not surprising that inhibition of NF-*κ*B and ERK1/2 pathways leads to block and reversal of EMT.

### 6.3. Noncoding RNA

The discovery of noncoding RNA unveiled a new layer of regulation of cellular function. Noncoding-miRNAs selectively bind mRNA, thus inhibiting their translation or promoting their degradation. Accumulating evidence shows that miRNAs regulate diverse biological processes, including cell proliferation, differentiation, and apoptosis [93, 94]. Recent studies have defined a large number of miRNAs associated with EMT and controlling the expression of EMT master transcription factors, suggesting a possible role also in peritoneal fibrosis.

In particular, miR29b and miR30A repress Snail1 expression [95, 96]. Moreover, members of the miR-200 family and miR-205 repress the translation of ZEB1 and ZEB2 miRNA. Notably, ZEB proteins repress the expression of miR-200 [97]. Interestingly, in hepatocyte cellular models Snail directly represses the miR-200c expression [98].

Studies on the role of noncoding RNA have been conducted mainly on experimental models of tumor EMT. Considering nontumoral experimental setting, studies have been performed mainly on experimental models of kidney and lung fibrosis. With regard to chronic progressive kidney disease, the roles of miR-21, miR-29, and miR-200 have been best established [99]. A mouse model of pulmonary fibrosis identified miR-31 as a direct modulator of integrin *α*5 and RhoA, proteins involved in migration and ECM deposition [100].

On the other hand, only a few data have accumulated so far on EMT/fibrosis of the peritoneum. PD-related peritoneal fibrosis is associated with a loss of miR-29b, and intraperitoneal delivering of plasmid expressing this miRNA in mice inhibited peritoneal fibrosis through an effect on TGF-*β*/Smad3 pathway [101]. Interestingly, expression of different miRNAs including miR-15a, miR-17, miR-21, miR-30, miR-192, and miR-377 from dialysis effluent correlated with peritoneal transport alterations in PD patients, suggesting a role of miRNA in PM damage [102]. In another study performed using MCs from effluent of patients undergoing PD, miRNA200c levels were found reduced in MC from PD patients [103]. A negative feedback mechanism involving TGF-*β*, miR-9-5p, NADPH oxidase 4 (NOX4), and playing a role in fibrosis of the mesothelial membrane has been recently described [104].

Besides miRNA, other noncoding RNAs (ncRNA) are abundantly transcribed in all cell types. Long noncoding RNAs can exert their effects on biological processes through a variety of mechanisms and can be involved in the pathophysiology of several diseases, including cancer and pulmonary fibrosis [105, 106]. Concerning peritoneal fibrosis, it has been recently reported that three lncRNAs target distinct mRNAs (Dok2, Ier3, HSP72, Junb, and Nedd9) involved in tissue inflammation and fibrosis [107]. Overall, the role of lncRNA in MCs EMT deserves future studies.

## 7. Conclusions

In the last years, the decrease in incidence rate of catheter complications coupled to the increased biocompatibility of dialysis solutions reduced the progressive damage to the PM during peritoneal dialysis. However, the incidence of peritoneal membrane problems remains high. To this purpose, current challenges are both the discovery of biomarkers (that could allow constantly monitoring the state of PM) and the understanding of molecular events underlying peritoneal damage in order to preserve or restore a peritoneal function. Thus, the study of molecular mechanisms involved in peritoneal fibrosis has both a basic and a translational relevance, appearing essential for the setting of more efficient therapies. Furthermore, it may conceivably be relevant in the possible treatment of other pathological conditions involving peritoneal fibrosis, such as postsurgical adhesions and peritoneal fibrosis induced by drugs, and peritoneal metastases [49, 108].

More efforts are needed to better elucidate the MCs molecular response to inflammatory/fibrogenic signals. Inhibition of main extracellular mediators as well as of specific players in the cascade of events triggered by TGF-*β* and by TLR/IL-1*β* could represent possible drugs that can simultaneously affect multiple target genes. Moreover, the possible control of the levels of particular ncRNAs, for example, by simple antagomiRs approaches, could conceivably guarantee the specific regulation of gene expression for more targeted therapies.
